# Respiratory Syncytial Virus (RSV) Update

**DOI:** 10.3390/v14102110

**Published:** 2022-09-23

**Authors:** Leonard R. Krilov, Norbert J. Roberts

**Affiliations:** 1 Department of Pediatrics, Division of Pediatric Infectious Diseases, NYU Long Island School of Medicine and NYU Langone Hospital-Long Island, Mineola, NY 11501, USA; 2 Division of Infectious Diseases and Immunology, Department of Medicine, New York University Grossman School of Medicine, New York, NY 10016, USA; 3 Division of Infectious Diseases, Department of Internal Medicine, University of Texas Medical Branch at Galveston, Galveston, TX 77555, USA

Since the initial identification of respiratory syncytial virus (RSV) in 1956, much has been learned about the epidemiological impact and clinical manifestations of RSV infections [1]. The overall burden of RSV can be assessed in a number of ways including hospitalizations; mortality in certain populations (e.g., elderly, developing countries); and medically attended lower respiratory illnesses [2]. Natural infection does not provide long-lasting immunity and reinfections with RSV occur throughout life [3]. Despite numerous studies documenting the major impact of RSV in infants, young children, the elderly, and other groups with chronic medical conditions, management remains primarily supportive and includes administration of supplemental oxygen, adequate hydration, and mechanical ventilation when needed. No vaccine is currently available.

Ribavirin was licensed by the Food and Drug administration in 1986 for the aerosolized treatment of severe RSV infection in hospitalized patients; however, due to inconclusive overall efficacy, potential toxicity, environmental exposure risk, inconvenient route of administration, and high cost, its use is extremely limited [4].

Immunoprophylaxis, with the humanized monoclonal antibody palivizumab, has been available since 1998 for high-risk infants (e.g., extremely premature babies and/or those with chronic lung disease of infancy or hemodynamically significant congenital heart disease), but cost concerns and uncertainty surrounding optimal indications have limited its use even for these groups [5]. In recent years, major advances in the understanding of the molecular structure of the virus and critical aspects of host responses to the virus are paving the way for major advances in treatment and prevention of RSV infections. Effective antiviral drug candidates, vaccines, and improved long-acting monoclonal antibodies are all in advanced development and clinical trials [6,7].

This is an exciting time in RSV research. With the recent advances in the understanding of the structure and function of the virus and host responses as well as ongoing further appreciation of the epidemiology of the infection in children and adults, we are on the cusp of major advances in the treatment and prevention of RSV disease. We hope this volume helps crystallize a number of these issues related to this important pathogen.

## Data Availability

Not applicable.
